# Short Report: First Outbreak of Atlantic Rainforest Spotted Fever in North‐Eastern of Brazil—Clinical Features and Eco‐Epidemiological Investigation

**DOI:** 10.1111/zph.70093

**Published:** 2026-09-15

**Authors:** Gabriel Nunes de Oliveira, Ruan Carvalho Passos, Ricardo Siqueira Bovendorp, Matheus Pasini‐Martins, Igor Silva Silito, Thiago Fernandes Martins, George Rego Albuquerque, Maiara Santos Zuchonelli, Carina Santos Pereira, Ana Clara de Carvalho Pereira, Lucas Monteiro Santos de Almeida, Rodrigo Nogueira Angerami, Marcelo Bahia Labruna, Anaiá da Paixão Sevá

**Affiliations:** ^1^ Programa de Pós‐graduação em Ciência Animal—PPGCA UESC (Universidade Estadual de Santa Cruz) Ilhéus Bahia Brazil; ^2^ Programa de Pós‐graduação em Ecologia—PPGECO UESC (Universidade Estadual de Santa Cruz) Ilhéus Bahia Brazil; ^3^ Departamento de Medicina Veterinária Preventiva e Saúde Animal, Faculdade de Medicina Veterinária e Zootecnia Universidade de São Paulo São Paulo São Paulo Brazil; ^4^ Seção de Epidemiologia Hospitalar, Hospital das Clínicas Universidade Estadual de Campinas Campinas São Paulo Brazil

**Keywords:** *Amblyomma ovale*, inoculation eschar, polymerase chain reaction, *Rickettsia parkeri*, serology, tick‐borne zoonosis

## Abstract

**Introduction:**

Since 2010, six cases of Atlantic rainforest spotted fever (ARSF) have been confirmed and reported in Brazil, a tick‐borne disease caused by *Rickettsia parkeri* strain Atlantic rainforest and associated with the tick *Amblyomma ovale* . So far, none of the cases have been associated with death. One of the cases occurred in 2018 in the municipality of Ilhéus, Bahia state, that is in Atlantic Forest Biome. In this study, we report the first confirmed outbreak of ARSF in Brazil, with three new cases associated with tick bites when entering Atlantic Forest areas in the same municipality, from March 11 to April 4, 2025.

**Methods:**

Scab samples from inoculation eschars of three patients presenting post‐tick‐bite symptoms were collected for molecular analysis targeting a fragment of the ompA gene of *R. parkeri*, followed by DNA sequencing. Paired serum samples (acute and convalescent phases) were evaluated by immunofluorescence assay (IFA) against *R. parkeri* antigens. Additionally, small mammals were captured using Sherman and Tomahawk traps during the same period in the exposure sites to evaluate tick infestation dynamics and species.

**Results:**

All patients developed symptoms 7–10 days after the bite, including inoculation eschar with progressive enlargement, exanthema, regional lymphadenopathy, fever, myalgia, and prostration; all responded to doxycycline treatment. Molecular analysis confirmed the presence of *R. parkeri* strain Atlantic rainforest DNA in all scab samples. Seroconversion (IgG) to *R. parkeri* was demonstrated in two patients. A total of 10 immature *A. ovale* ticks (larvae and nymphs) were collected parasitizing the wild cricetid rodents *Hylaeamys seuanezi*  (three individuals) and  *Thaptomys nigrita* (one individual) in the outbreak area.

**Conclusions:**

This report demonstrates three new confirmed cases and the first reported cluster of ARSF in Brazil, confirmed by laboratorial criteria, including molecular analyses, demonstrating the potential of the southern region of Bahia for transmission of *R. parkeri* , thus making it an endemic area for the disease.

## Impacts

- This article reports the first outbreak of Atlantic rainforest spotted fever in Brazil, with clinical, molecular and serological confirmation, and encounter of the proven vector of the bioagent parasitizing wild rodents.
- The integration of human cases with ecological findings reinforces a One Health perspective, demonstrating how interactions among humans, wildlife and vectors influence disease emergence, emphasizing the need to balance environmental conservation with safe human activities.
- The recognition of southern Bahia as an endemic area underscores the importance of strengthening health systems, promoting early diagnosis, environmental monitoring and coordinated actions across sectors to reduce health risks and support sustainable management of Atlantic Forest regions.

## 1 Introduction

Rickettsioses are zoonotic diseases caused by bacteria of the genus *Rickettsia*. In Brazil, *Rickettsia rickettsii* causes Brazilian spotted fever (BSF), which is a main concern among known tick‐borne diseases and is responsible for severe symptoms and a high risk of death (Campos et al. 2016; Mendes et al. 2024). Although BSF has been common in the country for several years, mainly concentrated in the South and South‐east Regions (Santos et al. 2023), the cases of Atlantic rainforest spotted fever (ARSF), caused by *Rickettsia parkeri* strain Atlantic rainforest, are uncommon and have been observed only in recent years (Blanton 2013; Albuquerque et al. 2025).

Reported since 2010 (Albuquerque et al. 2025; Krawczak et al. 2016; Martiniano et al. 2022; Sevá et al. 2019; Silva et al. 2011; Spolidorio et al. 2010), ARSF differs from BSF in that it is milder and, to date, there have been no reports of deaths. However, it presents symptoms such as inflammation with crusty lesions at the tick‐bite site (inoculation eschar), fever, exanthema, body aches, headache, prostration and regional lymphadenopathy (Faccini‐Martínez et al. 2014). *Rickettsia parkeri* strain Atlantic rainforest is primarily transmitted through the bite of ticks of the species *Amblyomma ovale* (Krawczak and Labruna 2018; Sevá et al. 2019), whose adult stage mainly parasitizes wild and domestic carnivores and accidentally infests humans, and the immature stages (larvae and nymphs) parasitize Cricetidae rodents (Jones et al. 1972; Rocha et al. 2022).

In Brazil, six laboratory‐confirmed cases of ARSF have been reported in four states, including São Paulo (Albuquerque et al. 2025; Spolidorio et al. 2010), Bahia (Sevá et al. 2019; Silva et al. 2011), Santa Catarina (Krawczak et al. 2016) and Rio de Janeiro (Martiniano et al. 2022). In the municipality of Ilhéus (Bahia), one case was reported in 2018, which was the second case for the state (Sevá et al. 2019) and the fourth in the country. In this study, we report an outbreak of three new cases of ARSF in an Atlantic rainforest area in the municipality of Ilhéus, occurring in 2025 within less than a month, and we identified the presence of the tick *A. ovale* in the same region and period infesting wild hosts.

## 2 Case Presentations

### 2.1 Area Where the Cases Occurred

Three patients were bitten by ticks when entering a ‘cabruca’ area (Atlantic rainforest agroforestry with cocoa) in the municipality of Ilhéus in eastern Bahia state, Brazil (Figure 1), on March 11, 2025 (Patient A), April 1, 2025 (Patient B) and April 4, 2025 (Patient C).

**FIGURE 1:**
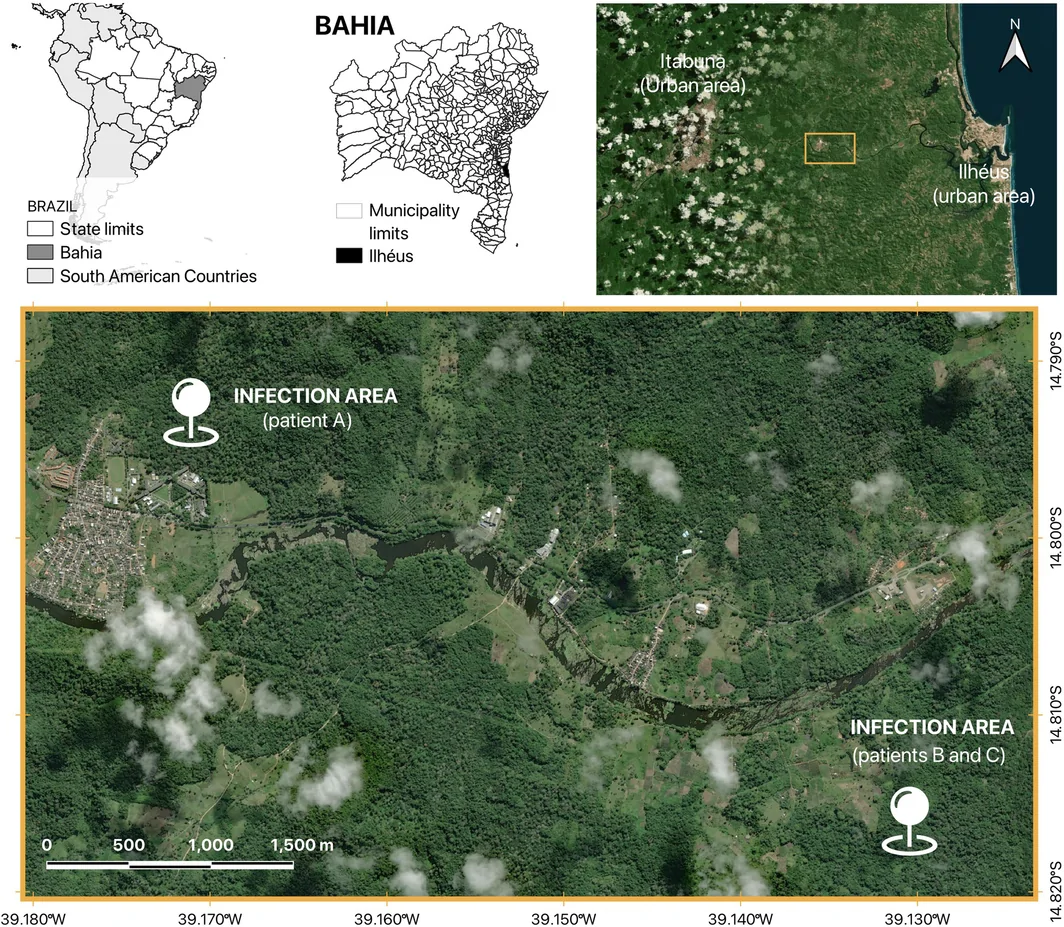
Site of infection (tick bite) in Patients A, B and C, in the municipality of Ilhéus, Bahia, Brazil.

### 2.2 Clinical Signs and Symptoms

#### 2.2.1 Patient A

An adult male, White race, 45 years old, was bitten by a tick on the wrist (March 11, 2025). The attached tick was removed manually after 1 day (time) of parasitism. On the second day after the bite (DAB), the site where the tick had attached was already erythematous. The redness and swelling at the bite site increased until seven DAB (March 18), when there was an eschar. On the 9th DAB (March 20), night fever began, and on the 11th (March 22) exanthema initially appeared on the arm at the bite site and progressed to a diffuse form. On the 14th DAB (March 25), the fever became daytime as well; there was generalized myalgia, headache and prostration, and treatment with doxycycline was initiated (100 mg orally every 12 h for 7 days). After the start of treatment, the prostration improved on the following day; fever and exanthema ceased on the third day of treatment. The crusty lesion, redness and oedema at the bite site remained for more than 10 days, regressing slowly and progressively, and healing was complete 15 days after the start of treatment.

#### 2.2.2 Patient B

An adult male, White race, 21 years old, observed on April 1, 2025, a dead tick on the right leg, above the pant leg. An area of redness and swelling resembling a tick bite was observed on the front of the right ankle (Figure 2) 3 days after the parasite was found (April 4). Between the 8th and 9th DAB (April 9) and, more markedly, on April 10, the patient developed a fluctuating fever (reaching up to 38°C), weakness and body aches. On the 10th DAB (April 11), treatment with doxycycline was started (100 mg orally every 12 h for 7 days), but lymphadenopathy appeared in the inguinal region, on the same side of the leg where the bite occurred, which became more painful over the next 2 days (11th and 12th DAB; April 12 and 13), to the point of hindering mobility while walking. During these days, the fever subsided and the weakness reduced. On the 13th DAB (April 14), lymphadenopathy became less painful and began to slowly and gradually subside. The bite eschar remained until the 17th DAB, along with the end of antibiotic use (April 18).

**FIGURE 2:**
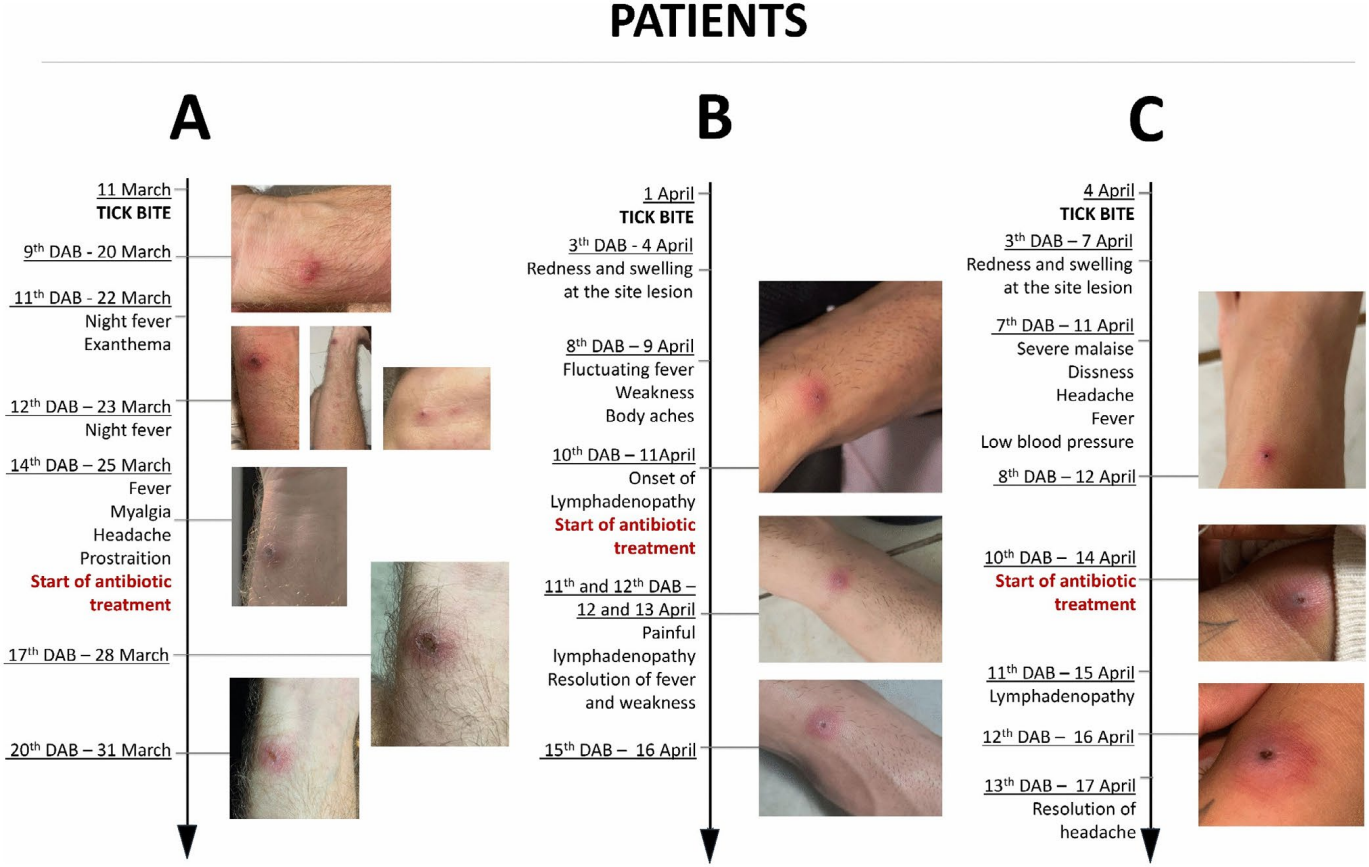
Chronology of the clinical course of Patients A, B and C after being bitten by ticks in an area of Atlantic rainforest in Ilhéus, Bahia, north‐eastern Brazil at year 2025.

#### 2.2.3 Patient C

An adult female, White race, 22 years old, was bitten by a tick on the lower part of her left leg (April 4, 2025). The tick was attached and was removed manually after an estimated period of 3 h attached to the skin. On the first day of the patient's environmental exposure. Redness and swelling were observed at the bite site 3 days later (April 7), with weak bite eschar evolution. On the 7th DAB (April 11), symptoms such as severe malaise, dizziness, headache, fever and episodes of low blood pressure began. On the 10th DAB (April 14), treatment with doxycycline was started (100 mg orally every 12 h for 7 days). On the 11th DAB (April 15), lymphadenopathy appeared in the inguinal region, on the same side of the leg where the bite occurred. The systemic symptoms disappeared the following day (April 16), with only residual headache remaining for another day (April 17). The lesion at the bite site healed slowly, remaining visibly open until at least 26 days after the tick bite (April 20).

All patients were examined by a physician who prescribed the treatment, and also, they reported that they discarded the ticks immediately after removing them from their bodies. The chronology of the clinical course of the three patients is illustrated in Figure 2, and the clinical signs and symptoms are listed in Table 1.

**TABLE 1:** Clinical signs and symptoms presented by Patients A, B and C after being bitten by ticks in an area of Atlantic rainforest in Ilhéus, Bahia, north‐eastern Brazil.

Clinical alteration	Patient A	Patient B	Patient C
Redness at the tick‐bite site	Yes	Yes	Yes
Swelling at the tick‐bite site	Yes	Yes	Yes
Inoculation eschar	Yes	Yes	Yes
Fever	Yes	Yes	Yes
Fluctuating fever	No	Yes	No
Malaise/prostration	Yes	Yes (weakness)	Yes (severe indisposition)
Myalgia/body aches	Yes	Yes	No
Headache	Yes	Not reported	Yes
Dizziness	No	No	Yes
Low blood pressure (hypotension)	No	No	Yes
Exanthema	Yes (arm and body)	No	No
Regional lymphadenopathy (inguinal)	No	Yes	Yes
Difficulty walking due to inguinal swelling	No	Yes	No
Slow‐healing lesion	Yes	Yes	Yes

### 2.3 Laboratory Diagnoses

From each patient, scabs were collected by biopsy, from the inoculation eschar of the tick‐bite site for molecular diagnosis. The scabs were collected from Patient A on March 22, 2025 (11 DAB; 3 days before the start of doxycycline therapy), from Patient B on April 11, 2025 (10 DAB; the same day doxycycline therapy began), and from Patient C on April 11, 2025 (7 DAB; 3 days before the start of doxycycline therapy). After collecting, the scabs from each patient were kept in 100% ethyl alcohol and stored at −20°C until laboratory analysis.

The molecular diagnostics and sequencing were performed in the laboratory of Parasitic Diseases of the Department of Preventive Veterinary Medicine and Animal Health, School of Veterinary Medicine and Animal Science, University of São Paulo, São Paulo, Brazil. In the laboratory, the scab (approximately 10 mg) from each patient underwent DNA extraction using the DNAeasy Blood and Tissue Kit (Qiagen, Valencia, CA, USA) and was tested by a polymerase chain reaction (PCR) protocol using primers Rr190.70F and Rr190.701R, targeting a 632‐bp fragment of the rickettsial 190‐kDa outer membrane protein gene (*ompA*), according to Roux et al. (1996). The *ompA* gene is known to be present only in *Rickettsia* species of the spotted fever group (SFG), which includes both pathogenic and non‐pathogenic agents (Stenos and Walker 2000). PCR reactions included positive (DNA of *Rickettsia vini*) and negative (sterile water) controls. Aliquots of PCR products were electrophoresed in 1.5% agarose gel. When bands at a height compatible with 632 nucleotides were present, the PCR product was treated with ExoSAP‐IT (USB, Cleveland, OH, USA) and sequenced in both directions (forward and reverse) in an ABI automated sequencer (ABI Prism 3500 Genetic; Applied Biosystems, Foster City, CA, USA). The consensus sequence from each patient was submitted to BLAST analyses (National Center for Biotechnology Information 2025) to infer closest identities in GenBank (Altschul et al. 1990).

Paired blood samples for serologic analyses were collected from the three patients, as follows: Patient A sera collected at 17 and 31 DAB; Patient B sera collected at 14 and 27 DAB; and Patient C sera collected at 11 and 24 DAB. Sera samples were tested by immunofluorescence assay (IFA) against crude antigens of four SFG‐*Rickettsia* species from Brazil, namely *R. parkeri* strain At24, *R. rickettsii* strain Taiaçu, *Rickettsia amblyommatis* strain Ac37 and *Rickettsia rhipicephali* strain HJ5, as previously described (Sevá et al. 2019; Sousa et al. 2024). Briefly, sera were diluted in twofold increments with phosphate‐buffered saline, starting from the 1:64 dilution. Slides were incubated with fluorescein isothiocyanate‐labelled goat anti‐human IgG (Sigma, St. Louis, MO, USA). The end point IgG antibody titre reacting with each of the four *Rickettsia* antigens was determined for each serum sample.

#### 2.3.1 Results of Diagnostic

PCR on scab DNA of the three patients yielded amplicons of the expected size, and after DNA sequencing, they generated three consensus sequences (556 base pairs [bp] after excluding the primer sequences from the flanks) that were 100% identical to each other. By BLAST analysis, this consensus sequence was 100% identical (556/556 bp) under 100% query cover to an *ompA* partial sequence of *R. parkeri* strain Atlantic rainforest (MN027565), previously reported in another patient from Ilhéus (Sevá et al. 2019), and then 99.8% identical (555/556 bp) under 100% query cover to several sequences of *R. parkeri* strain Atlantic rainforest from other regions of Brazil (JQ906784, MK962698, MF536975) and other Latin American countries (ON148412, CP040325, MF034495, OP375582). The present partial *ompA* sequence has been submitted to GenBank under accession number PZ193058.

Serological analyses revealed that the first serum samples from the three cases were non‐reactive to SFG *Rickettsia* at the 1:64 screening dilution. In contrast, the second serum samples were reactive, as follows: Patient A had endpoint titres of 128 to *R. parkeri* and 64 to both *R. rickettsii* and *R. amblyommatis*. Patient B had endpoint titres of 64 to *R. parkeri, R. rickettsii* and *R. amblyommatis*. Patient C had endpoint titres of 512 to *R. parkeri*, 128 to *R. rickettsii* and 64 to *R. amblyommatis*. All sera were non‐reactive to *R. rhipicephali* . Given that a serum that is non‐reactive at a dilution of 1:64 would most likely be reactive at a dilution of 1:32, the current findings indicate that Patients A and C seroconverted to *R. parkeri* , as their second serum samples had endpoint titres that were at least four times higher than those of the first serum samples. In contrast, Patient B did not seroconvert to any of the four rickettsial antigens.

### 2.4 Field Study

During the period from November 2024 to April 2025, at the same location where the three patients were bitten by ticks, a field study was being conducted to capture small mammals to investigate other infectious agents and tick infestations related to other studies. With the unexpected occurrence of the three clinical cases in humans at the end of this period, it was decided to analyse whether ticks collected from small mammals corresponded to a known vector of *R. parkeri* . From November 2024 to April 2025, in each of the two areas indicated in Figure 1, small mammals were captured using Sherman traps (small: 7.62 × 8.89 × 22.86 cm) and Tomahawk traps (50 × 21.5 × 20 cm), baited with a mixture of peanut flour, palm oil, banana and cornmeal. The traps were arranged in a grid pattern over an area of 200 m^2^, consisting of two transects 20 m apart, with traps placed every 10 m along each transect. The traps were placed alternately in terms of type and arrangement on the ground and in the understory. In Patient A's area, 40 traps were used, distributed across two collection sub‐areas, while in Patients B and C's areas, 60 traps were used, distributed across three sub‐areas. This variation in the total number of traps was due to differences in size, environmental heterogeneity and extent of the areas investigated, thus ensuring proportional sampling effort and the possibility of obtaining a comparable inventory of small mammal fauna at each site.

The traps were set up and left available for capture for five consecutive nights each month, alternating between the locations where the cases occurred. The total sampling effort was 1500 trap‐nights, with 600 trap‐nights in Patient A's area and 900 trap‐nights in Patients B and C's areas. They were checked every morning in the early hours of the day. The captured individuals were physically restrained and then chemically restrained with ketamine (marsupials 20 mg/kg, and rodents 75 mg/kg intramuscular) and xylazine (marsupials 10 mg/kg and rodents 5 mg/kg, intramuscular), and then identified by species and age group, and examined for tick infestations. When present, ticks were collected with the help of tweezers, placed into microtubes containing 70% alcohol and stored at room temperature until sent to the laboratory for species identification following Marques et al. (2004), Barbieri et al. (2008) and Barros‐Battesti et al. (2024).

The collection of small mammals has been approved by the Ethics Committee on Animal Use of the State University of Santa Cruz (CEUA/UESC) under No. 022/24 and by the Biodiversity Authorization and Information System of Brazil–SISBIO No. 83348‐4.

#### 2.4.1 Data From Field Study

Due to the low density of small mammals in the study area (R.S. Bovendorp, unpublished data), only 11 animals were captured and sampled, corresponding to four marsupials of the Didelphidae family and seven rodents of the Cricetidae family (Table 2). Of the 11 small mammals examined, seven (64%) were infested with a total of 28 ticks, being of two species: *Amblyomma ovale* , found on four mammals (36%) and comprising seven larvae and three nymphs; and *Ixodes loricatus* , found on five mammals (45%), represented by 10 larvae and eight nymphs. Marsupials were infested solely by *I. loricatus*, whereas rodents were infested by both *A. ovale* and *I. loricatus*. Notably, all three captured individuals of the rodent *Hylaeamys seuanezi* were infested by *A. ovale* ticks.

**TABLE 2:** Ticks collected from small mammals in two areas of an Atlantic rainforest agroforestry with cocoa in the municipality of Ilhéus, Bahia state, Brazil, from October 2024 to March 2025.

Small mammals				Ticks		
Species	Sex	Area^a^	Date	*Amblyomma ovale*	*Ixodes loricatus*	Total
**Marsupials**						
*Didelphis aurita*	♂	A	March 14, 2025		5♂, 3♀, 1 nymph	9
*Marmosa murinae*	♀	A	March 12, 2025		5 nymphs	5
*Marmosops incanus*	♂	A	November 22, 2024			0
*Monodelphis americana*	♂	A	November 22, 2024			0
**Rodents**						
*Euryoryzomys russatus*	♂	BC	December 6, 2024			0
*Hylaeamys seuanezi*	♀	BC	October 4, 2024	2 nymphs		2
*Hylaeamys seuanezi*	♂	A	November 22, 2024	5 larvae, 1 nymph	1 nymph	7
*Hylaeamys seuanezi*	♀	A	November 22, 2024	1 larva	1 nymph	2
*Rhipidomys mastacalis*	♂	A	November 22, 2024		2 larvae	2
*Rhipidomys mastacalis*	♀	A	January 28, 2025			0
*Thaptomys nigrita*	♀	A	November 22, 2024	1 larva		1
**Total**	11			10	18	28

^a^ Area A includes the place where Patient A acquired tick infestation, whereas Area BC includes the places where Patients B and C acquired tick infestations (see Figure 1).

## 3 Discussion

This study corresponds to the seventh, eighth and ninth laboratory‐confirmed and published cases and the first reported outbreak in Brazil of rickettsiosis caused by *R. parkeri* strain Atlantic rainforest, the so‐called ARSF in the municipality of Ilhéus where a single case was previously described (Sevá et al. 2019; Silva et al. 2011). The clinical features of presented cases in the outbreak, including presence of inoculation eschar, a mild disease and benign evolution observed were similar to those described in previous cases of *R. parkeri* spotted fever reported in Brazil (Albuquerque et al. 2025; Krawczak et al. 2016; Martiniano et al. 2022; Sevá et al. 2019; Silva et al. 2011; Spolidorio et al. 2010), except for one patient who presented with dizziness and hypotension. The incubation period of the cases reported here ranged from 4 to 11 days, an interval already reported in the literature (Faccini‐Martínez et al. 2018; Silva‐Ramos et al. 2021).

Differently of the observed in the spotted fever caused by *R. rickettsii* , where the risk of progression to severe disease and high case fatality rate are expected when specific treatment is started after the fourth day of symptoms (Biggs et al. 2016), the three cases in this outbreak did not present with severe clinical manifestations or organ dysfunction, despite the slow healing of the inoculation eschars (Figure 2). In this report, patients were treated with doxycycline, which is the antimicrobial of choice for the treatment of rickettsiosis, regardless of age or clinical severity (Portillo et al. 2017). Patients showed clinical improvement after the first few days of antibiotic treatment, corroborating existing literature that fever and inflammatory symptoms tend to disappear between 24 and 72 h after the start of therapy (Ministério da Saúde, Secretaria de Vigilância em Saúde e Ambiente 2017; Portillo et al. 2017).

The ARSF cases were confirmed according to the criteria set out in the Ministry of Health Technical Manual (CTMS) No. 88/2025 (Ministério da Saúde, Secretaria de Vigilância em Saúde e Ambiente 2025). Initially, patients were classified as suspected cases, characterized by sudden onset of fever, headache and myalgia, associated with a history of tick bites and contact with wild animals (Ministério da Saúde, Secretaria de Vigilância em Saúde e Ambiente 2025). Confirmation was obtained through molecular tests, which generated DNA sequences of *R. parkeri* strain Atlantic from scabs collected from the inoculation eschar of the three patients. In addition, two cases (Cases 1 and 3) were also confirmed by seroconversion to *R. parkeri* antigens. These results fulfill the laboratory and clinical‐epidemiological criteria defined by the CTMS.

The clinical case of Patient B, despite being confirmed by molecular analysis, was not confirmed by seroconversion, since the titre of the second serum sample was only 64 for SFG‐*Rickettsia* spp. IFA is the most widely used serological method for the diagnosis of spotted fever and is considered the gold standard (Ministério da Saúde, Secretaria de Vigilância em Saudé e Ambiente 2017; Portillo et al. 2017). In general, antibodies are detectable from the 7th to 10th day of infection (Portillo et al. 2017). The absence of seroconversion in Patient B as well as seroconversion with relatively low titres in Patients 1 (128) and 3 (512), may be related to the early initiation of treatment with doxycycline, which can reduce the detectable immune response, as reported for different *Rickettsia* species with larger case series worldwide (Biggs et al. 2016; Portillo et al. 2017). In previous reports of human infection by *R. parkeri* strain Atlantic rainforest, in which patients received doxycycline between 2 and 7 days after the onset of fever, seroconversion to *R. parkeri* occurred with final titres of 128 (Silva et al. 2011), 512 (Spolidorio et al. 2010) and 1024 (Krawczak et al. 2016; Sevá et al. 2019). This study reinforces the ease of collecting and preserving viable material for the detection of *R. parkeri* by PCR (Krawczak et al. 2016), minimizing the limitations of indirect immunofluorescence serology in distinguishing species from the spotted fever group and in potential situations where seroconversion does not occur. This serological condition reinforces that the diagnosis of such an infection should not be based solely on serology, because even though it is relatively quick, it does not present good reliability. Although there are no reports of death caused by this disease, the symptoms evolve rapidly and, therefore, rapid treatment is necessary. Thus, in these cases, clinical‐epidemiological diagnosis can be fundamental to quickly stopping the symptoms. However, molecular and serological diagnoses are still important, as they can serve as a foundation to elucidate the dynamics of transmission and contribute to future rapid diagnoses and treatments.

It is interesting to note that the 556 bp‐fragments of the *ompA* gene of *R. parkeri* generated from the three patients in this study were 100% identical to a single sequence from GenBank (MN027565), which was generated from another patient who contracted ARSF in the same study region in 2018 (Sevá et al. 2019). This partial *ompA* sequence differs from the other *ompA* sequences of *R. parkeri* strain Atlantic rainforest from GenBank by just one SNP (single nucleotide polymorphism: C → A), which results in the substitution of a Histidine (CAT codon) for an Asparagine (AAT codon). Although the number of confirmed cases of ARSF is still low in the literature (Silva‐Ramos et al. 2021), future studies should explore the genetic variations of different *R. parkeri* isolates and their possible clinical and eco‐epidemiological implications.

The *A. ovale* tick is recognized as the vector of *R. parkeri* strain Atlantic rainforest in Brazil (Silva‐Ramos et al. 2021; Patiño‐Quiroz et al. 2026). In the first case of ARSF reported in 2018 in the same area as the present study, the vector tick was properly identified as a female *A. ovale* (Sevá et al. 2019). The fact that we found *A. ovale* larvae and nymphs in rodents suggests that there is an established population of this tick species in this area. Considering the current epidemiological knowledge of ARSF in the country, as well as the report of immature *A. ovale* in some rodents sampled in the area, it is possible to hypothesize that the three cases reported here may be associated with parasitism by *A. ovale* infected with *R. parkeri* strain Atlantic rainforest in the patients. In a previous study conducted in the Ilhéus region, in areas adjacent to the present study, Rocha et al. (2022) captured 609 small mammals of 12 different species during the years 2015–2017. Similarly to the present study, the authors reported that marsupials were infested only by *I. loricatus* , while *A. ovale* was the predominant species infesting small rodents. Furthermore, the Cricetidae *H. seuanezi* was the most abundant species and also the most parasitized by immature stages of *A. ovale* . In addition, the authors detected DNA from *R. parkeri* strain Atlantic rainforest in some of the *A. ovale* collected from rodents. Although this study involved a much smaller number of captured rodents, it corroborates the results reported by Rocha et al. (2022). Thus, it is possible that the rodent *H. seuanezi* is the main host species for immature *A. ovale* in the Ilhéus region. Future studies should assess whether this rodent also functions as an amplifier host of *R. parkeri* strain Atlantic rainforest for *A. ovale* , as was found by Krawczak and Labruna (2018) for the Cricetidae *Euryorysomys russatus* on the southern coast of the state of São Paulo, south‐eastern Brazil.

Although we did not collect adult stage of *A. ovale* on wild and domestic carnivores or on human patients, it should not be a strong limitation to understanding of the disease's transmission dynamics. A recent study conducted in the same rural area of Ilhéus reported that 15% of *A. ovale* collected from domestic dogs were infected with *R. parkeri* SARF, in addition to serological evidence of past infection by *R. parkeri* in some of these animals (de Oliveira et al. 2019). To complete the picture of a potential parasite cycle in the region, we must consider that the infection sites of the three patients are part of a large forest area that shares its edges with anthropized areas, and consequently with domestic dogs, such as farms, and, especially, the site of patient A is bordered by a village. In both scenarios, the domestic dog is kept in a domicile and frequently has free access to the extra‐residential environment, including this forest. Furthermore, because these areas are ‘cabruca’, humans frequent the interior of the forest, sometimes with their dogs, as already observed at the region (Silva et al. 2022; dos Santos et al. 2017). Furthermore, wild canids also frequent this region of the Atlantic Forest (Sánchez‐Lalinde et al. 2019). These findings and now the description of the first outbreak of ARSF reinforce the existence of a potentially endemic natural focus of the pathogen, with a continuous risk of transmission to humans in the municipality. This result strengthens the hypothesis that peridomestic dogs, by frequenting fragments of the Atlantic Forest and returning parasitized, contribute to the maintenance of infection in the peridomestic environment (Ministério da Saúde, Secretaria de Vigilância em Saúde e Ambiente 2025).

Additionally, despite mild systemic clinical manifestations and a benign course, this study demonstrates the need for early suspicion, timely treatment and qualified laboratory investigation (including the collection of appropriate biological material) to understand the true epidemiological scenario of ARSF in the country (Faccini‐Martínez et al. 2021).

Finally, the description of the outbreak reinforces the need for the population and health professionals in areas known to have ARSF transmission to be aware of the local epidemiological scenario, potentially risky exposures, and disease characteristics so that early suspicion and qualified care of suspected cases can be provided.

## 4 Conclusion

This study is the first report of an ARSF outbreak, confirming three new cases in Brazil. Adding this find with a previous case, and also with the active and persistent presence of the *A. ovale* vector in the region, it is possible to consolidate the municipality of Ilhéus as an established transmission area. Laboratory confirmation, associated with the finding of immature *A. ovale* parasitizing wild rodents, shows that the essential elements of the ecological cycle remain present and operative in the environment. Given this scenario, from a One Health perspective, it is essential to maintain continuous epidemiological and acarological surveillance, especially in rural and agroforestry areas and zones of interface between the Atlantic Forest and human dwellings. In addition, community education and risk communication actions are urgently needed to guide residents, rural workers and visitors on prevention, early recognition of symptoms, and prompt seeking of medical care and appropriate treatment. Finally, the results demonstrate not only the ecological relevance of the hosts and vectors involved, but also the need for intersectoral responses to reduce human exposure and strengthen local capacity for detection, prevention and case management. These findings contribute to the regional understanding of ARSF and reinforce the importance of integrating public health, environmental management and social awareness in addressing the disease.

## Funding

This work was supported by Universidade Estadual de Santa Cruz (073.6762.2024.0031848‐95).

## Ethics Statement

This study was approved by the Ethics Committee on Animal Use of the State University of Santa Cruz (No. 022/24) and SISBIO (No. 83348‐4).

## Consent

Written informed consent for publication of clinical details and clinical image was obtained from the patient.

## Conflicts of Interest

The authors declare no conflicts of interest.

## Data Availability

The data that support the findings of this study are available from the corresponding author upon reasonable request.
